# Screening for *Astragalus hamosus* Triterpenoid Saponins Using HPTLC Methods: Prior Identification of Azukisaponin Isomers

**DOI:** 10.3390/molecules27175376

**Published:** 2022-08-23

**Authors:** Khouloud Nafti, Geraldine Giacinti, Sonia Marghali, Christine Delgado Raynaud

**Affiliations:** 1 Laboratoire de Chimie Ago-Industrielle (LCA), Université de Toulouse, INRAe, 4 Allée Emile Monso, 31030 Toulouse, France; 2 Laboratoire de Génétique Moléculaire, Immunologie et Biotechnologie, Faculté des Sciences de Tunis, Université de Tunis El Manar, Tunis 1068, Tunisia; 3 Centre d’Application et de Traitement des Agro-Ressources (CATAR), Toulouse-INP, ENSIACET, 4 Allée Emile Monso, 31030 Toulouse, France

**Keywords:** *Astragalus hamosus*, HPTLC, triterpenoid saponin, soyasaponin, azukisaponin

## Abstract

Due to their particular structural characteristics, the extraction and isolation of saponins from plants present a serious challenge. In this study, specific extraction protocols were first implemented to extract the secondary metabolites from *Astragalus hamosus* and, more precisely, the saponins. Subsequent purification of the extracts was based on a single chromatographic technique, high-performance thin-layer chromatography, applying two development systems: a one-step system that separated molecules according to their polarity and a multiple development system that made it possible to detect the triterpenoid saponins, azukisaponin or soyasapogenol at a retarded Rf of 0.2. The difficulties of detecting the *Astragalus hamosus* saponins encountered during the extraction and purification of the extracts have been highlighted and the strategy carried out to isolate the saponins has been discussed.

## 1. Introduction

Plants are a valuable source of a wide range of secondary metabolites which are used as pharmaceuticals, agrochemicals, flavors, fragrances, dyes, biopesticides, and food additives. In the early 19th century, many sensitive ingredients were isolated and introduced into medical practice. *Astragalus* L. is one of the largest genera of flowering plants in the Fabaceae family. As annual or perennial grasses, *Astragalus* L. plants are widely distributed in temperate and arid regions. Until now, it is estimated that the genus contains from 2000 to 3000 species and more than 250 taxonomic sections worldwide [1].

*Astragalus hamosus* L. is a prostrate or ascending annual or biennial herbaceous plant distributed throughout Southern Europe, the Mediterranean, the Caucasus, and Central and Southwest Asia. This plant is encountered in the form of a herb, with paripinnate or odd-pinnate leaves. Its inflorescence is racemose, the umbels are spike shaped or solitary, and the peduncles are generally axillary. Its flowers are bracted; bracteoles are present. Its stamens are diadelphous, without vexillary stamens, and the anthers are uniform. It has a sessile or pedunculated ovary. Its fruits are sessile with two valves that are unilocular or partially or totally bilocular by an intrusive membrane. Its seeds are often reniform [2].

*Astragalus hamosus* is one of the plants belonging to the genus *Astragalus* that has been used in herbal and traditional Indian and Iranian medicine. Hachim et al. and Shojaii et al. demonstrated the significant anti-inflammatory activity of alcoholic extracts of *Astragalus* *hamosus* pods in animal models [3,4]. In addition, Western blot analysis of cyclooxygenase-2, interleukin-1, and tumor necrosis factor showed that *A. hamosus* has enhanced effects against the neuroinflammation caused by Aß in a rat model of Alzheimer’s disease [5]. The recent work of Mahmoodi et al. highlighted the anti-proliferative effects of *Astragalus hamosus* plant extract on breast cancer cells [6]. 

In recent years, advances in research on *Astragalus* species have been made because of their polyphenol [7,8], and saponin content. *Astragalus* species contain both cycloartane and oleanane saponins, structures which are based on soyasapogenol B as aglycone [9].

Saponins are secondary metabolites widely distributed throughout the plant kingdom. They act as a chemical barrier or shield in the plant defense system against pathogens and herbivores [10]. Saponins are a vast group of glycosides that are widely distributed in higher plants. Their surface-active properties are what distinguish these compounds from other glycosides. They dissolve in water to form colloidal solutions that foam upon shaking [11]. Saponins are polar molecules consisting of a triterpene or steroid aglycone with one or more sugar chains. They are one of the largest and most diverse groups of natural plant products [12]. The saponins are incompletely soluble in two different solvents, one hydrophilic and the other hydrophobic [13].

Discoveries of the biological activities of saponins have not been limited to traditional uses only, but also more recently in pharmaceutical applications [14]. These molecules have hemolytic [15,16], molluscicidal, anti-inflammatory, antifungal or antiyeast, antibacterial or antimicrobial, antiparasitic, antitumor and antiviral [11], anticancer [17,18,19], and antioxidant activities [20,21,22], and can also act as an immunological adjuvant [23].

In *Astragalus hamosus*, a mixture of two saponins showed antineoplastic activity against two breast carcinoma cell lines (estrogen receptor MCF-7 (ER)-positive and MDA-MB 231-ER-negative) [24,25]. Two saponins of the oleanane type, Peregrinozide I and Azukisaponin V of *A. hamosus*, have shown dose-dependent modulation of lymphocyte proliferation in four cancer types: BC1 (human breast cancer), Lu1 (human lung cancer), Col2 (human colon cancer), and LNCaP (human prostate cancer) [26]. 

The importance of saponins as pharmaceutical agents, especially in the fight against cancer, has led to the invention of new extraction methods in order to obtain the maximum yield to meet the growing demand [27]. Since saponins typically occur in plants as a mixture of structurally related forms with very similar polarities, their separation remains a challenge [28]. For this reason, it is generally necessary to combine several techniques (e.g., TLC, column chromatography, flash chromatography, Sephadex chromatography, and HPLC) to obtain pure compounds for determination of the structure and the biological activity [28,29]. In addition, the absence of a chromophore prevents their confirmation and quantification by UV [30]. 

Another problem that can also be pointed out is that the content of saponins is easily affected by the geographical location, cultivation method, harvesting phases, and many other factors, all of which lead to unqualified quality, reduced biological activity, and limited clinical applications. Given the challenges of determining saponins, researchers around the world have proposed different analysis methods for saponins [31].

Very few phytochemical studies have been carried out on *Astragalus hamosus* and, to date, only one has been published on Tunisian *Astragalus hamosus,* however, this only considered the morphology [2]. 

The HPTLC screening method has the advantages of multisampling analysis with a great capacity of charge and a multiplicity of mobile phases, which make it a simple, fast, efficient, and stable separation technology [32]. Moreover, HPTLC has become one of the main methods for determining saponins in natural medicines and preparations [33], and gives results comparable with those of HPLC [34].

About 70% of the studies carried out on saponin extraction are based on conventional technologies such as maceration, Soxhlet, and reflux extraction, and only 30% are based on green technologies. In this study, conventional methods were applied ranging from the simplest to the more complex in order to screen the total metabolites of *Astragalus hamosus* and to identify the saponins of this plant [27,35]. The extracts obtained were purified and analyzed by HPTLC according to a simple development system that separated the metabolites according to their polarity and a multiple development system that allowed the detection of triterpene saponins. 

This study reports the investigation conducted on *Astragalus hamosus* and describes the strategy carried out to isolate the saponins, which is extremely difficult because of their amphiphilic properties. Different extraction protocols adapted from the literature were investigated to target the saponin fractions, and the HPTLC polarity mobile phases were developed and optimized to enhance the isolation of the molecules. It is the first time that chemical screening of *Astragalus hamosus* saponins from Tunisia has been reported.

## 2. Results and Discussion

### 2.1. Extraction of Compounds from Astragalus homosus 

As expected, extractable yield differed in terms of the extraction protocol used (Table 1). The greatest difference is observed between methanol extraction and the other protocols used to try to be more selective in the recovery of saponins. The extraction yield decreased between the methanol extract (M1) and Protocols P1 to P4, demonstrating the initial selectivity of compounds by the choice of the protocol extraction. Protocol M1 was a simple methanolic extraction of the ground and dried plant material, whereas Protocols P1 to P2 used several organic solvents of different polarities in addition to water: −0.77 < logP < 3.5.

Due to their amphiphilic properties, saponosides are usually extracted with water or alcohols using many different techniques [27,36]. It has been demonstrated that butanol and methanol are the best solvents for the extraction of triterpenoid saponins from gac seeds [37]. Butanol has also been reported to be the solvent of choice for the extraction of saponins from the shell of *Chenopodium quinoa* seeds [36], while methanol has been widely used to extract saponins from a wide range of plant matrices [14,27]. Methanol (logP = −0.77) used at 85% was the most polar of the organic solvents selected in this work and allowed us to extract not only saponins but also all molecules with hydrophilic characteristics [38]. However, if saponins represent the most bioactive molecules of interest in *Astragalus*, they are not abundant. The protocol P1 was based only on methanol (logP = −0.77)–butanol (logP = 0.84) extraction, whereas P2 to P4 first used organic solvents such as hexane (logP = 3.9), chloroform ((logP = 2), ether petroleum (logP = 0.84), or ethyl acetate (logP = 0.73) to remove lipophilic molecules before final fractionation with butanol, as in P1 [39,40,41]. 

As the plant from the population of Zaghouan presented the highest methanolic extraction yield, all the extraction protocols were first evaluated with this raw plant material.

### 2.2. Preliminary Evaluation of the Abundance of Secondary Metabolites for Screening Triterpenoid Saponins by HPTLC 

**Figure 1:**
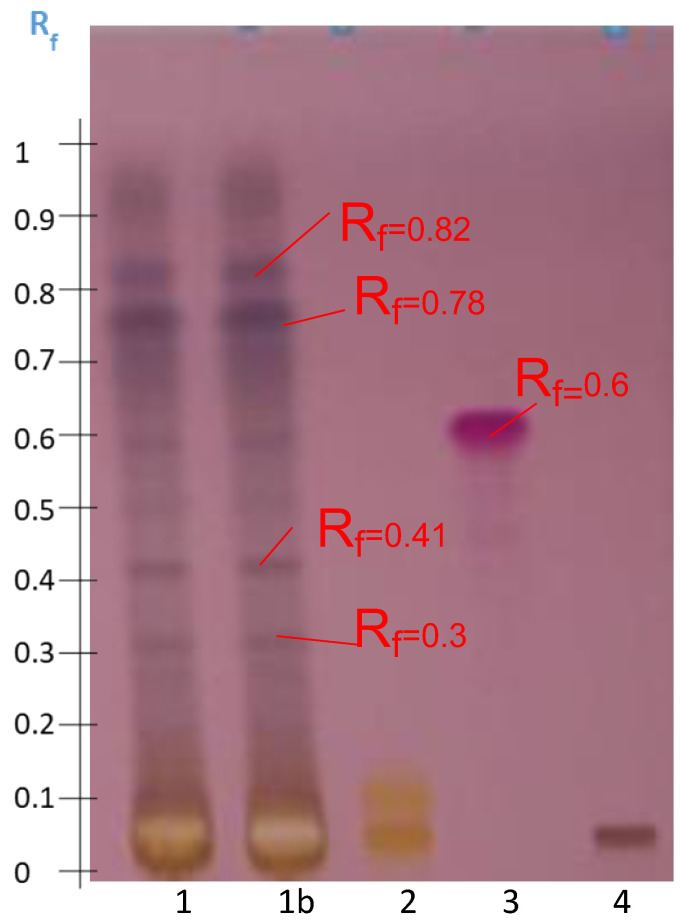
HPTLC profile obtained after migration and visualization with anisaldehyde-H_2_SO_4_ of the Bizerte extract according to Protocol M1 (Tracks 1 and 1b) and compared with the standards: solanine (Track 2), senegenin (Track 3), and digitonin (Track 4). Elution system S1.

The preliminary HPTLC analysis provided initial information on the abundance of secondary metabolites present in the methanolic extract and on the separation and detection power of the first development system selected, S1. Eight spots could be observed and were retarded at different Rf values (0–0.85) on the first plate (Figure 1). This analysis was compared with standards for the three families of saponins, i.e., a steroidal saponoside, digitonin, a glycol-alkaloid saponin, solanine, and an aglycone triterpenoid saponin, namely senegenin. Solanine and digitonin, which are glycosylated, remained at Rf = 0, while the aglycone senegenin migrated to Rf = 0.6 because of its lower polarity and higher affinity with the elution solvent.

**Figure 2:**
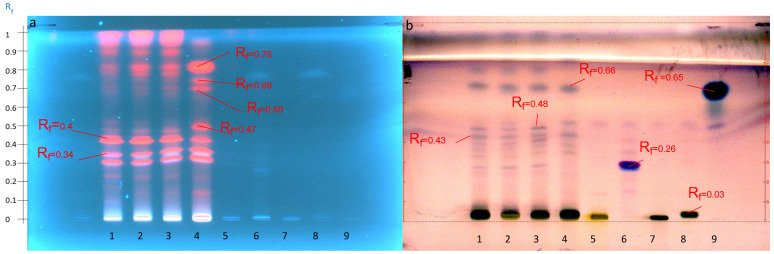
HPTLC profiles of the four extracts of the four populations obtained according to the M1 extraction method compared with the standards: (**a**) before visualization at 366 nm and (**b**) after visualization with anisaldehyde-H_2_SO_4_ under white light according to the S1 elution system. Track 1, Bizerte; Track 2, Kairouan; Track 3, Zaghouan; Track 4, Siliana; Track 5, solanine; Track 6, senegenin; Track 7, digitonin; Track 8, glucose-fructose; Track 9, oleanolic acid.

The extraction method M1 was then compared among the four selected populations of *Astragalus hamosus* (Figure 2). The extract from the population of the Siliana region seemed to be enriched in certain methanol-extractable compounds. Four more intense spots appeared at 366 nm then, after visualization with anisaldehyde-H_2_SO_4_, they were retarded at Rf values, 0.15, 0.46, 0.66, 0.72, and 0.80. Glycosylated standards, solanine, digitonin, and glucose–fructose sugars remained at the deposit spot (Rf = 0) but aglycone-type standards such as senegenin and the triterpenoid oleanolic acid were migrated. As all selected standards were detectable only after visualization with anisaldehyde-H_2_SO_4_ under white light and not under UV at 366 nm, the spots before and after derivatization cannot be associated with saponin molecules. Only the derivatization step could highlight molecules of the saponin family that do not absorb UV at 366 nm. Conversely, some spots that could be detected under UV at 366 nm no longer appeared after derivatization, namely the spots at Rf = 0.72 and Rf = 0.8. These results showed that saponin molecules from the extracts probably remained at the sample deposit on the HPTLC plate and if aglycones were present in the methanol extract, they coeluted with other molecules. The molecules seem to be coeluted at the same Rf of 0.66 as oleanolic acid in all the extracts. 

### 2.3. Optimization of the Saponin Fraction Purification of Astragalus hamosus

**Figure 3:**
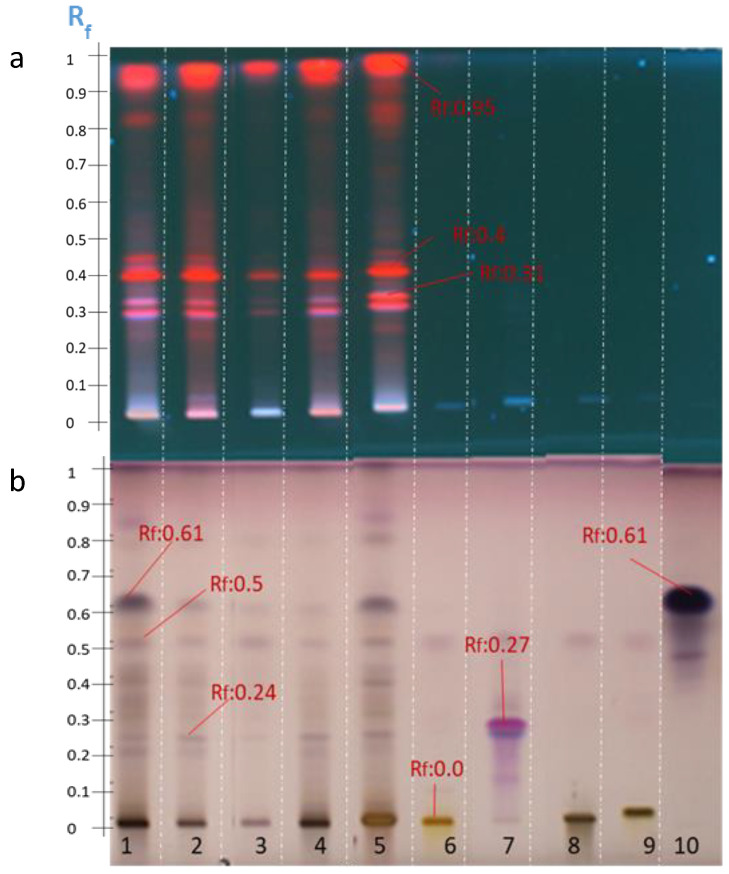
HPTLC profile obtained at 366 nm before visualization (**a**) and after visualization with anisaldehyde-H_2_SO_4_ (**b**) of the 4 Zaghouan extracts according to the S1 elution system and P1 (Track 1), P2 (Track 2), P3 (Track 3), P4 (Track 4) and compared with M1 (Track 5) and the standards: Track 6, solanine; Track 7, senegenin; Track 8, digitonin; Track 9, glucose-fructose; Track 10, oleanolic acid.

**Figure 4:**
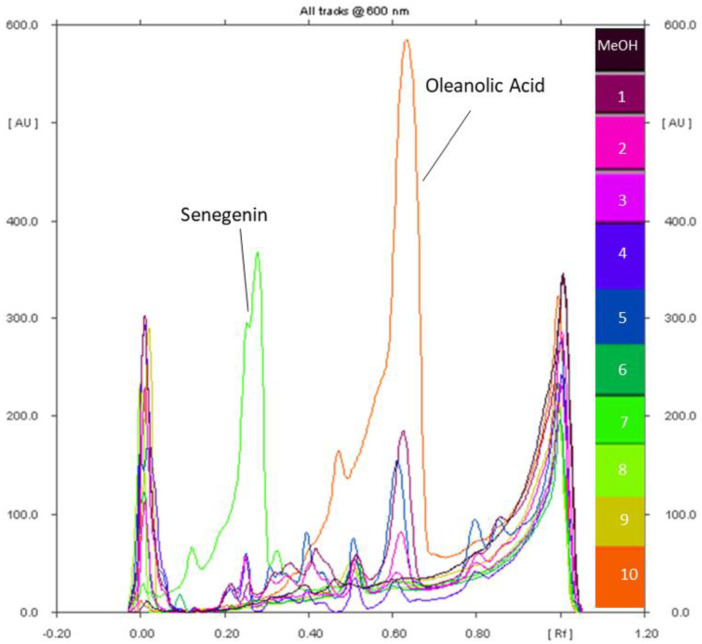
The densitometric chromatograms of the Zaghouan extracts (Tracks 1–5) and the standards (Tracks 6–10): solanine (Track 6), senegenin (Track 7), digitonin (Track 8), glucose-fructose (Track 9), and oleanolic acid (Track 10) at 600 nm after visualization with anisaldehyde-H_2_SO_4_.

**Figure 5:**
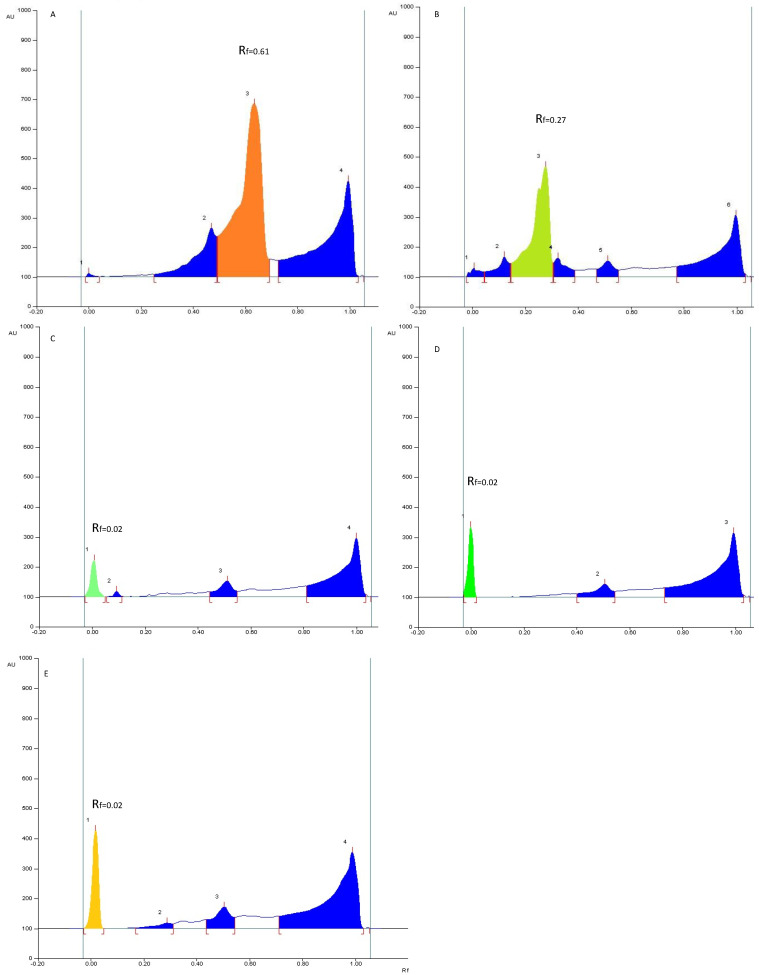
Densitograms of oleanolic acid (**A**), senegenin (**B**), solanine (**C**), digitonin (**D**), and glucose–fructose (**E**) at 600 nm after visualization with anisaldehyde-H_2_SO_4_.

**Figure 6:**
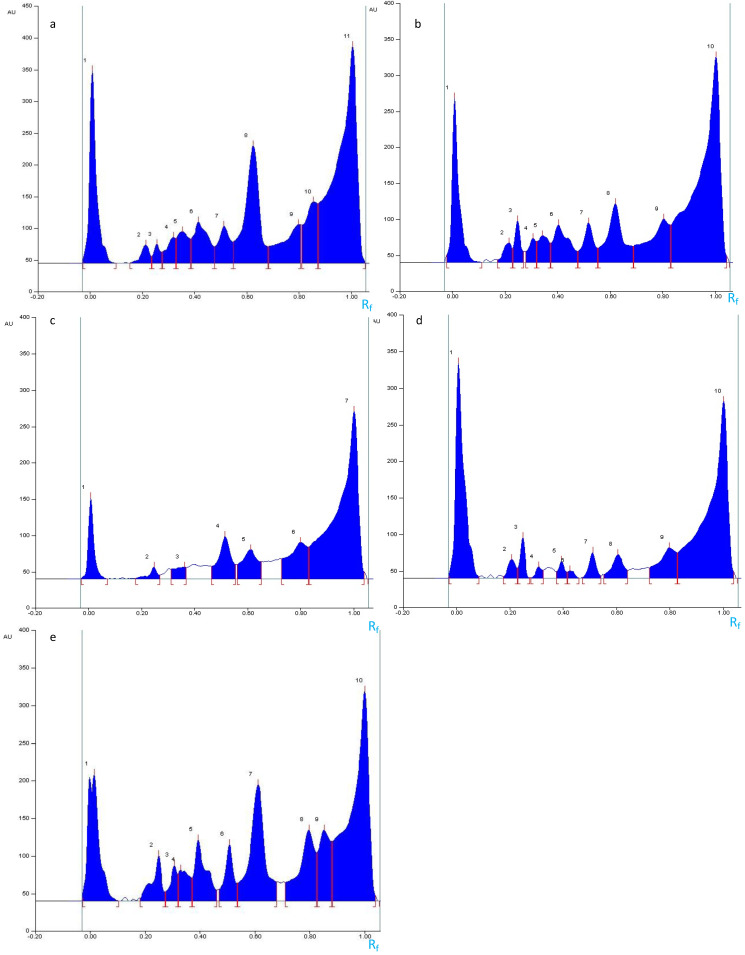
Densitograms of the extracts from the Zaghouan population obtained by P1 (**a**), P2 (**b**), P3 (**c**), P4 (**d**), and M1 (**e**): sequential extracts at 600 nm after visualization with anisaldehyde-H_2_SO_4_.

Since the single-step methanolic fractionation revealed the presence of many molecules in the HPTLC analysis, other purification protocols were explored as shown in Table 1. As the population of Zaghouan presented the most important yield of methanol extractable, this plant was selected as a model matrix for screening of the multi-step extraction protocols (Figure 3, Figure 4, Figure 5 and Figure 6).

The preliminary delipidation with hexane before the methanolic extraction was carried out by the P2 extraction protocol which allowed to increase the concentration of some metabolites and thus to improve their detection at 366 nm (Rf = 0.31; Rf = 0.40; and Rf = 0.95) (Figure 3). However, these molecules, while also being detectable after visualization with anisaldehyde-H_2_SO_4_, remained at small quantities and disappeared in the P3 extract.

In contrast, as expected, the P3 fractionation protocol seemed to be more selective than the other ones. Densitogram 3 in Figure 6 shows the elimination of peaks compared with other densitograms. P3 is based on the successive elimination of fractions with organic solvents of increasing polarity (logP(hexane) = 3.5 < logP(chloroform) = 2.0 < logP(ethyl acetate) = 0.8). Two hypotheses can be proposed: either the final fraction had a lower concentration of chemical compounds and the detection threshold of such molecules was reached, or it was effectively more selective and the coeluted compounds were removed. The amounts deposited were subsequently increased for future optimization analyses. 

The protocol P4 also used organic solvents of weaker polarity than ethanol or methanol which were used after the initial ethanol extraction, this allowed the elimination of some compounds compared with P1 or M1 as shown in Figure 3 and Figure 6. 

After visualization with anisaldehyde-H_2_SO_4_, the four extracts presented similar HPTLC bands, with densitograms of different intensities at Rf = 0.24, Rf = 0.40, Rf = 0.50, and Rf = 0.61.

Compounds eluted at Rf = 0.61 were quantitatively reduced in the P2, P3, and P4 extracts, whereas compounds retarded at Rf = 0.4 seem to have been eliminated by the P3 purification protocol. Triterpenoid saponins, such as oleanolic acid, were apparently detected at Rf = 0.66 in all extracts and needed to be confirmed.

### 2.4. Optimization of the Elution Systems

#### 2.4.1. Disclosure of Terpenoids and Preliminary Detection of Soyasaponin Using the Multiple Development System (S2)

The use of several elution systems made it possible to successively separate the molecules according to their affinity for eluents of increasing polarity.

**Figure 7:**
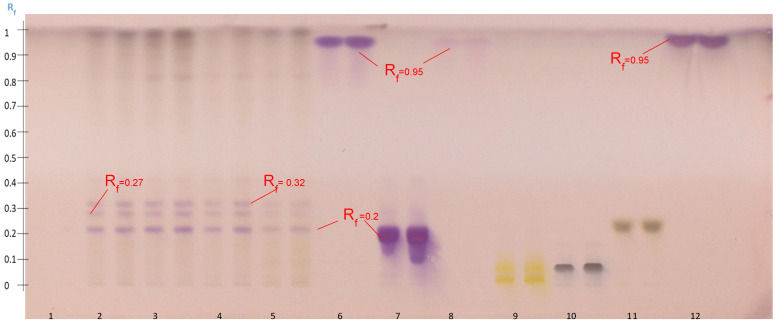
HPTLC profile obtained after visualization with anisaldehyde-H_2_SO_4_ of the four Zaghouan extracts according to P1 (Track 2), P2 (Track 3), P3 (Track 1), and P4 (Track 4), and according to the M1 method (Track 5) as well as the standards: soyasapogenol (Track 6), soyasaponin (Track 7), senegenin (Track 8), solanine (Track 9), digitonin (Track 10), glucose–fructose (Track 11), and oleanolic acid (Track 12), according to the S2 elution system).

**Figure 8:**
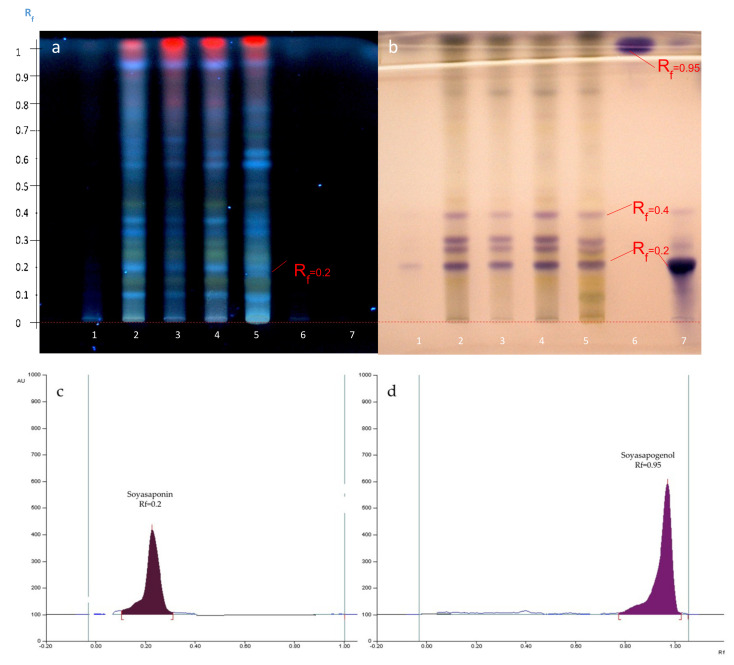
HPTLC profile of the four Zaghouan extracts obtained before visualization under UV at 366 nm (**a**) and after visualization with anisaldehyde-H_2_SO_4_ (**b**), according to P1 (Track 2), P2 (Track 3), P3 (Track 1), and P4 (Track 4), and according to the M1 method (Track 5) as well as the standards: soyasapogenol (Track 6) and soyasaponin (Track 7), using the S2 elution system. (**c**) Densitogram of soyasaponin at 600 nm at Rf = 0.2 and (**d**) densitogram of soyasapogenol at 600 nm at Rf = 0.95 according to the S2 elution system.

The initial results showed that the S1 system could not elute the saponins, which remained at an Rf value of around 0. Saponins are polar molecules and the stationary phase, silica, is also polar [42]. A second elution with a more polar system (S2) than the previous one highlighted new spots detectable at an absorbance wavelength of 366 nm as a result of the purification of some bands that were already detectable at this wavelength (Figure 7 and Figure 8). Twelve spots were detected from Rf = 0 to Rf = 0.9. All the molecules which were revealed by red spots and which were numerous in the previous HPTLC analyses with the S1 system seemed to have migrated to the solvent front. Consequently, the new elution system allowed the migration of the polar molecules which remained at the line of deposition in the previous analyses. The increase in the eluting force in the S2 system from SS1 to SS3 allowed the system to elute the less polar molecules up to the solvent front and retarded the most polar molecules at an Rf value of 0.2, as observed after visualization with anisaldehyde-H_2_SO_4_ (Figure 8b).

The HPTLC analysis of the Zaghouan extracts (P1—P4, M1) were then compared with the specific saponin standards of *Astragalus*, i.e., soyasaponin, an isomer of azukisaponin already identified in *Astragalus hamosus* [7,22], and its aglycone soyasapogenol in addition to the previous standards (solanine, senegenin and digitonin) used specifically to develop the organic solvent extraction method. Soyasaponin was detected at Rf = 0.2 with an intense purple spot, while oleanolic acid, soyasapogenol, and senegenin were detected by two intense purple spots at Rf = 0.95. As expected, the saponin standards were undetectable at 366 nm.

The presence of a spot retarded at the Rf value of 0.2 with the same Rf value as soyasaponin after visualization with anisaldehyde-H_2_SO_4_ suggested the presence of the saponins however some molecules were also detected at 366 nm at this same retarded Rf value for all extracts, except for the P3 Zaghouan extract (Figure 8a).

The profile of the Zaghouan extract obtained according to P3 showed no spot after depositing 10 µL (Track 1, Figure 7) however after the volume was increased to 25 µL (Track 1, Figure 8b), a single purple spot at Rf = 0.2 was detected after development and only a single blue spot at Rf = 0 (Figure 8a) was observed at 366 nm. This extraction protocol ultimately appears to be the most selective protocol for purifying saponins. 

#### 2.4.2. Tentatively Identified Triterpenoid Saponins in the A. hamosus Populations from Bizerte, Siliana and Kairouan

**Figure 9:**
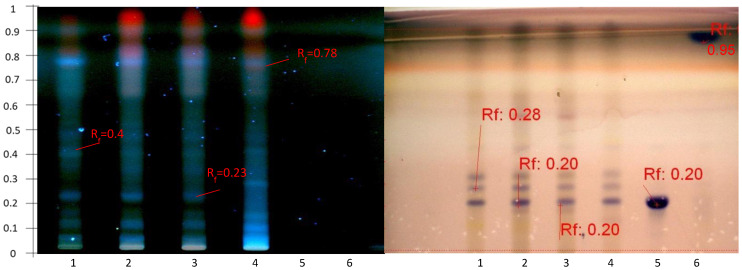
HPTLC profile of the four populations obtained after visualization with anisaldehyde-H_2_SO_4_: Zaghouan (Track 1), Siliana (Track 2), Bizerte (Track 3), and Kairouan (Track 4) according to P1 as well as the standards: soyasaponin (Track 5) and soyasapogenol (Track 6) using the S2 elution system.

The HPTLC profiles of the Bizerte, Siliana, and Kairouan populations obtained according to P1 after visualization with an anisaldehyde-H_2_SO_4_ reagent were similar (Figure 9). In fact, three major purple spots were detected: the first spot at the same Rf as soyasaponin (Rf = 0.2), a second at Rf = 0.28, and a third one at Rf = 0.32. The spot retarded at an Rf value of 0.4 in the Zaghouan extract observed in Figure 8b was much less intense in this analysis. Soyasapogenol was detected as usual at Rf = 0.95.

Saponins do not contain conjugated double bonds that can form chromophores, and are therefore difficult to detect under UV [43,44,45,46,47]. The use of a developer such as sulfuric anisaldehyde [48], allowed the saponins to take on blue, yellow, green, or purple colors [49,50]. 

To conclude, in this step, triterpenoid saponins are most likely to be detected at the same Rf as soyasaponin after visualization with anisaldehyde-H_2_SO_4_ reagent, with a higher intensity in the Siliana population than in the Bizerte and Kairouan populations.

### 2.5. Hydrolysis of A. hamosus Saponin Extracts

**Figure 10:**
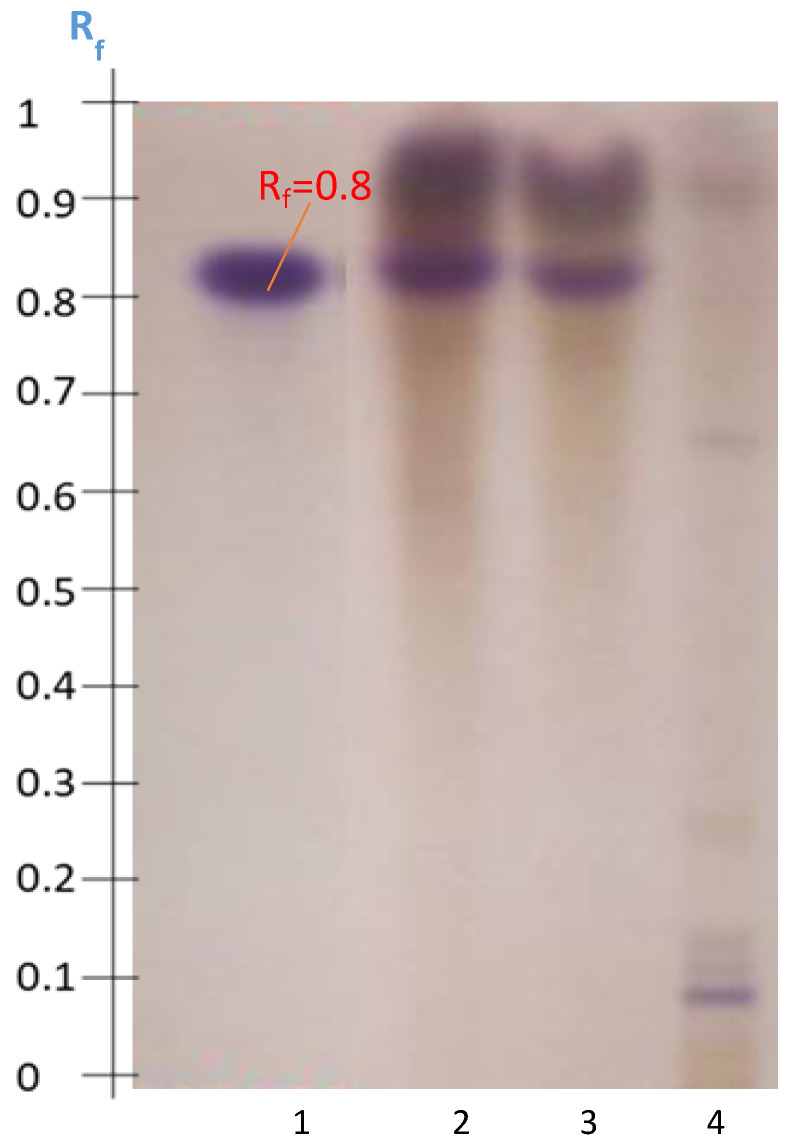
HPTLC profile obtained after visualization with anisaldehyde-H_2_SO_4_ using the SS2 system. Chloroform phases of the hydrolyzed soyasaponin standard (Track 1) and of the hydrolyzed Zaghouan-P3 extract (Tracks 2 and 3) and the Zaghouan-P3 extract that was not hydrolyzed (Track 4).

As demonstrated, protocol P3 seems to be the most selective for extracting triterpenoid saponins from *Astragalus hamosus*. P3 extracts of the Zaghouan population were hydrolyzed at the same time as the soyasaponin standard. The HPTLC profile obtained after hydrolysis confirmed the presence of the saponin (Figure 10). In Tracks 1 and 2 only one spot was retarded at an Rf value of 0.80 as seen for the standard hydrolyzed on Track 3. The SS2 system was used rather than SS3 in order to manage the migration of the aglycone after hydrolysis before the solvent front.

These results suggest the presence of soyasaponin or its isomer azukisaponin or even both in the Zaghouan-P3 extract. The HPTLC-MS^2^ coupling should be carried out to confirm the identification of these saponins in *Astragalus hamosus* populations from Zaghouan, Bizerte, Siliana, and Kairouan.

### 2.6. Quantification of Soyasapogenol

The quantification of saponins carried out by HPTLC was compared with the results found by spectrophotometry. The quantification of saponins by HPTLC was based on the analysis of the soyasapogenol content after hydrolysis of the extract and isolation of the molecule after migration on the silicate plate. The Zaghouan-P3-hydrolyzed extract presented 33 mg/g of soyasapogenol compared with 56 mg/g equivalent soyasapogneol obtained by spectrophotometry. The HPTLC method allowed us to focus the quantification on the targeted saponin, whereas overall saponin-like molecules were quantified by spectrophotometry. About 59% of the soyasaponin (equivalent soyasapogenol) could represent the saponin fraction of the Zaghouan-P3-hydrolyzed extract.

## 3. Materials and Methods

### 3.1. Reagent, Chemical and Samples

All reagents and chemicals used in this study were of analytical grade. 

The plant was harvested in March 2019 in Tunisia from four different geographical locations in the north of the country: Zaghouan (36.3632487° N, 9.8998366° W), Siliana (36.0914446° N, 9.5667337° W), Bizerte (37.2529341° N, 9.7477197° W), and Kairouan (35.8476563° N, 9.5932661° W) (Figure S1). *Astragalus hamosus* was identified by Pr. A.Zoghlami (Institut National de la Recherche Agronomique de Tunisie (INRAT), Tunis, Tunisia), a specimen of each population (22101, 22102, 22103, and 22104) was deposited at the Laboratoire de Chimie Agro-industrielle (LCA, Université de Toulouse, INRAE, Toulouse, France), and a sample is available on request. All the plant material was dried in the open air and then ground to a fine powder in a slide blender. The fine powder from each population was stored in an airtight container away from light at room temperature.

### 3.2. Extraction Methods

**Figure 11:**
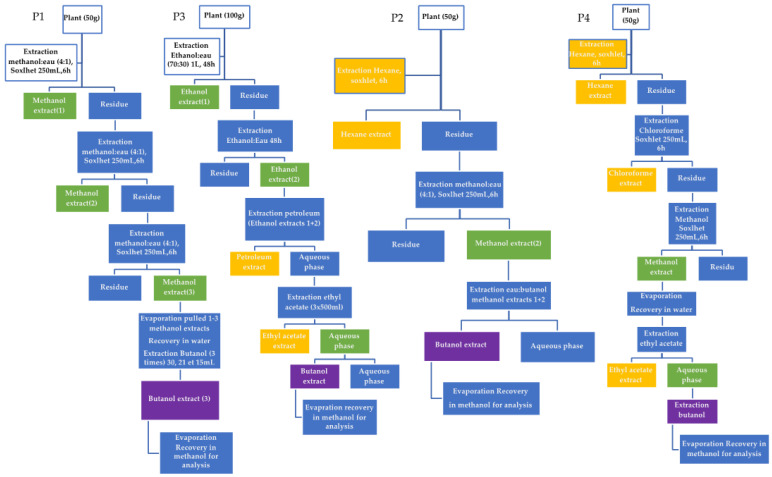
Diagrams of the extraction protocols from P1 to P4. Preliminary delipidation was carried out with hexane in the P2 and P4 protocols.

Five extraction protocols were chosen from the literature and adapted to extract saponins from *Astragalus hamosus*, ranging from a simple one-step protocol (M1) to more complex multi-step protocols (P1, P2, P3, and P4) (Figure 11).

The first liquid–solid extraction method adapted from Khakimov et al. [51], was implemented for the 4 populations of *Astragalus hamosus* from the 4 geographical locations: Bizerte, Siliana, Kairouan, and Zaghouan.

For this process, 25 mg of powder from each population was mixed with 1.5 mL of 85% MeOH in an Eppendorf tube, then heated to 100 °C and stirred in a vortex alternately for 5 minutes. This step was followed by centrifugation at 15,000× *g* for 3 min. The supernatant was recovered and kept at −20 °C before analysis.

The P1 extraction protocol has been adapted from the work of Ma et al. [52]. For this, 50 g of powder from each population was extracted and underwent 3 Soxhlet extraction cycles for 6 h with 250 mL of MeOH/H_2_O (4:1). The 3 extracts obtained were combined and evaporated to dryness and then added to 25 mL of hot distilled water at 70 °C. Next, 3 liquid–liquid extractions of the aqueous phase with 30, 21, and 15 mL successively were carried out. The 3 fractions obtained were combined, filtered, and evaporated to dryness at 50 °C.

For the other extraction protocols implemented (P2—P4), only the Zaghouan population was used. The P2 extraction protocol is that of Kambouche et al. [53]: 50 g of powder was extracted successively with 250 mL of hexane, then with 250 mL of MeOH. After removal of the solvent under a vacuum, the methanol residue was dissolved in water and re-extracted with 9 mL of n-BuOH.

The extraction protocol P3 is that of Maamria et al. [54]. One hundred grams of the Zaghouan population was macerated twice for 48 h in 1 L of EtOH/H_2_O (70:30). The recovered 2 L were filtered, dried under evaporation, and placed in 25 mL of distilled hot water. Next, 3 successive liquid–liquid extractions took place with 500 mL of petroleum ether, 500 mL of ethyl acetate 3 times, and then 500 mL of BuOH 3 times. The butanolic extract was kept for analysis. The extraction protocol P4 is that of Pistelli et al. [55]. Fifty grams of the Zaghouan population was extracted 3 times in a Soxhlet device with 250 mL of n-hexane, 250 mL of CHCl_3,_ and 250 mL of MeOH successively. The *n*-hexane and CHCl3 extracts were preserved and frozen. The methanolic extract was filtered and evaporated, then placed in 100 mL of distilled water. Subsequently, a liquid–liquid extraction of this aqueous phase was carried out with 70 mL of ethyl acetate. The resulting ethyl acetate extract was stored and frozen and the aqueous extract was extracted again with n-butanol. The butanolic extract obtained was kept cold for analysis.

### 3.3. High-Performance Thin-Layer Chromatography (HPTLC) Equipment and General Procedure

A CAMAG HPTLC system (CAMAG, Muttenz, Switzerland) consisting of a sample applicator from a TLC scanner and a visualizer were used for the analyses. All instruments were controlled via the WinCats 1·4·2 Planar Chromatography Manager (CAMAG) software platform. Silica gel plates (HPTLC 60 W F254, 10 × 10 cm, and HPTLC 60 W F254, 20 × 10 cm; Merck, Darmstadt, Germany) were used and developed in a horizontal double chamber CAMAG of 20 × 10 cm.

The samples were applied to silica plates with the ATS3 CAMAG autosampler (Muttenz, Switzerland). Two different development systems were chosen: (i) System S1 (30 mL of chloroform, 15 mL of hexane, and 5 mL of methanol) [56], and (ii) System S2 (SS1, SS2, and SS3) [57]:

− SS1: Dichloromethane and MeOH (92: 8) up to 26 mm for methylxanthines;
− SS2: EtOAc, toluene, formic acid, and H_2_O (8.7: 1.3: 1.7: 0.4) up to 70 mm for phenolic compounds;
− SS3: EtOAc, toluene, formic acid, and H_2_O (9: 1: 2.5: 1) up to 70 mm for saponins.

For Systems S1 and S2, developments were carried out in conventional glass chambers or in an automatic development chamber (ADC) (CAMAG, Muttenz, Switzerland) as follows: pre-dosing volume, 1000 nL; excess volume, 5000 nL; retraction volume, 100 nL; delivery speed, 150 nL s^−1^; filling speed, 500 nL s^−1^; rinsing time, 10 s; compression volume, 300 nL; compression time, 10 s; decompression volume, 240 nL. The first application position, X, was set at 15 mm and the application position Y was set at 10 mm. The distance between the tracks was calculated automatically from the number of deposits. The spray application mode was used with a band speed of 5 mm s^−1^ and a start delay of 50 ms. The length of the tape was set at 4 mm. Photos of non-derived plaques were taken at 366 nm using a visualizer (CAMAG, Muttenz, Switzerland). Photos of the derived plates were taken under white light. Assessments (during development of the solvent systems) were performed visually, checking and comparing the colors and retardation factor values (Rfs) of the spots that were visible in the photos taken at different wavelengths. Scans were taken using the TLC Scanner 4 (CAMAG, Muttenz, Switzerland) to obtain the spot spectra and generate the track chromatograms. All evaluations of the photos and scans were performed with visionCATS (CAMAG, Muttenz, Switzerland).

For derivatization, the plates were immersed in a solution of anisaldehyde-H_2_SO_4_ with a CAMAG TLC Immersion Device III (Muttenz, Switzerland) at an immersion rate of 2 cm s^−1^ for 2 s. Then plates were dried in an oven at 100 °C for 8 min or 10 min.

The plates were scanned at wavelengths between 200 and 500 nm before derivatization, and at 500 and 600 nm after derivatization with anisaldehyde-H_2_SO_4_ in a TLC Scanner 3 SC3 CAMAG, with deuterium and tungsten lamps (Muttenz, Switzerland). The slit size was set to 4 × 0.1 mm, the scan speed to 5 mm.s^−1^, and the data resolution to 50 m per step. Remission and absorption were selected as the type and mode of measurement, respectively. A second-order optical filter was used and the detector mode and sensitivity were automatic. 

The values of the retardation factor Rf were evaluated as the position of the substance relative to the position of the solvent front measured from the application position of the sample. The autosampler and HPTLC scanner, and the data acquisition and processing were controlled with the WinCats 1.4.6. 2002 Planar Chromatography Manager from CAMAG.

The 4 extracts of the 4 populations (Zaghouan, Siliana, Bizerte, and Kairouan) obtained according to M1 were analyzed by depositing 10 µL of each and 10 μL of the standards solutions at 1 g/L solanine, senegnin, and digitonin. Elutions were performed by S1 (Figure 1 and Figure 2) and also by S2 for the Zaghouan extract (Figure 8). The plates were scanned at 366 nm under UV before chemical derivatization and after chemical derivatization under white light with anisaldehyde-H_2_SO_4_. 

Next, 10 μL of the Zaghouan extracts obtained according to P1, P2, P3, and P4, and M1 were deposited twice on a cover plate, corresponding to deposits of 0.05 mg of the material, and 10 µL of solanine, senegnin, and digitonin was deposited twice (0.01 mg of the material). In addition to the saponin standards, the triterpenoid oleanolic acid was added, at a concentration of 1.02 g/L; 10 μL was deposited (0.01 mg). A mixture of glucose-fructose at a concentration of 0.5 g/L was added and 10 μL (0.005 mg) was deposited twice (Figure 3). The plate used was a 20 × 10 cm silica gel. The elution was carried out according to the S1 development system and detection was via visualization with sulfuric anisaldehyde. 

Next, 10 μL of the Zaghouan extracts obtained according to P1, P2, P3, and P4 (0.05 mg of material) and 25 μL of the Zaghouan-P3 extract were deposited for analysis (Figure 7 and Figure 8). For the standards: 10 µL of Soyasaponin B at a concentration of 1 g/L (0.01 mg of material); 5 µL of soyasapogenol at a concentration of 1.86 g/L (0.0093 mg of material); 10 µL of senegenin, solanine, digitonin, or a glucose–fructose mixture (1 g/L); and finally 10 µL of acid oleanolic at a concentration of 1.02 g/L (0.01 mg of material) were deposited. The elution was carried out according to the S2 development system. The detection was carried out after derivatization with sulfuric anisaldehyde under white light and before derivatization under UV at 366 nm. 

Next, the extracts from all populations (Zaghouan, Siliana, Bizerte, and Kairouan) obtained by the P1 protocol were deposited using the S2 development system. For this, 10 μL (5 g/L, 0.05 mg of material) of each extract was sampled. For the standards, 10 µL of soyasapogenol (1 g/L, 0.01 mg of material) and 5 µL of soyasaponin (1.86 g/L, 0.0093 mg of material) were added. The detection was achieved by visualization with sulfuric anisaldehyde under white light and under UV at 366 nm before derivatization (Figure 9).

### 3.4. Hydrolysis of Extracts by Acetyl Chloride

One milliliter of the Zaghouan extract (15 g/L) obtained using Protocol 3 was placed in an Eppendorf tube with 50 μL of acetyl chloride. The mixture was incubated for 1 h at 100 °C and then evaporated to dryness under nitrogen. After evaporation, 1 mL of water and 500 μL of chloroform were added and vortexed. After decantation, the chloroform phase was analyzed by HPTLC. The same procedure was applied to a standard solution of soyasaponin (1.86 g/L) in which 15 µL of the chloroform phase was deposited for each sample hydrolyzed on a silicate plate in addition to the non-hydrolyzed Zaghouan-P3 extract (20 µL). Elution was carried out following SS1 and SS2 only (Figure 10).

### 3.5. Quantification of Soyasapogenol in Extracts for Densitometry 

#### 3.5.1. Quantification of Sayasapogenol by Spectrophotometry

The quantitative determination of the soyasapogenol of the Zaghouan-P3 extract before and after hydrolysis was carried out according to the method of Ncube et al. [58], modified for a 96-well microplate: 25 µL of the sample or a solution of soyasapogenol at different concentrations, 25 µL of vanillin (8%), and 250 µL of sulfuric acid (72%) were added. The plate was incubated at 45 °C for 10 min and allowed to cool and the reading was taken at 544 nm. The device used was BMG-Labtech Spectrostar-Nano. The quantity of saponins was calculated according to DO calibration equation as a function of the concentration.

#### 3.5.2. Quantification of Sayasapogenol for Densitometry

**Figure 12:**
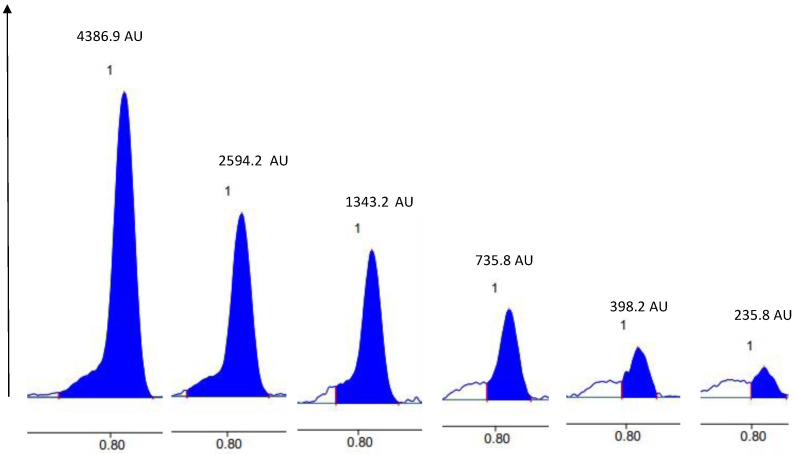
Densitograms of the reference soyasapogenol obtained at 6 different concentrations after visualization with anisaldehyde-H_2_SO_4_ and SS2 development, which were used to calculate the calibration curve.

A range of different concentrations of soyasapogenol (2 µL (1 g/L, 0.5 g/L, 0.25 g/L, 0.125 g/L, 0.0652 g/L and 0.0312 g/L)) were deposited on a 10 × 10 silica gel plate as well as two replicates of the chloroform phase of the hydrolyzed Zaghouan-P3 extract (15 µL) and Zaghouan-P3 extract before hydrolysis (25 µL) and the soyasaponin standard (5 µL of a 1.73 g/L). After SS2 elution and visualization with sulfuric anisaldehyde, the calibration curve was calculated from the peak areas of the 6 different concentrations of the reference soyasapogenol (Figure 12). The correlation coefficient R^2^ was 0.9929 and the equation used was y = 2156.6x + 200.45.

## 4. Conclusions

A strategy combining different extraction protocols and HPTLC elution systems was proposed in order to highlight the presence of saponins in four *Astragalus hamosus* populations from Tunisia. The various protocols implemented to extract the specific saponin fraction from the plants and to identify these molecules have made it possible to put forward a hypothesis for the identification and quantification of the azukisaponin and/or soyasaponin isomers. The multiple development system allowed for the detection of triterpenoid saponins at a retarded Rf of 0.2. These two molecules differ only in the presence of galactose in soyasaponin instead of glucose in azukisaponin and their differentiation is impossible by such analytical techniques. Nevertheless, the results of this study demonstrated that isomers are probably present in the different extracts of *Astragalus hamosus* from the Tunisian populations and that one of the four extraction protocols evaluated proved to be strongly effective in purifying *Astragalus hamosus* triterpenoid saponins. The HPTLC-MS^2^ coupling should be carried out to confirm the identification of these saponins in *Astragalus hamosus* populations from Zaghouan, Bizerte, Siliana, and Kairouan.

## Figures and Tables

**Table 1:** Results of extraction yields (DW/DW) for Protocols M1 and P1, P2, P3, and P4.

Extraction Protocol	Population of *A. hamosus*	Extraction Yield (DW/DW%)
M1	Zaghouan Siliana Bizerte Kairouan	11.5 ± 0.7 7.2 9.3 9.4
P1	Zaghouan Siliana Bizerte Kairouan	6.3 8.1 8.0 6.3
P2	Zaghouan	4.5
P3	Zaghouan	4.8
P4	Zaghouan	4.5

## Data Availability

The data presented in this study are available on request to the corresponding author.

## Supplementary Materials

The following supporting information can be downloaded at: https://www.mdpi.com/article/10.3390/molecules27175376/s1, Figure S1: Four populations of Astragalus hamosus collected in Tunisia. Populations of (a) Bizerte, (b) Kairouan, (c) Siliana and (d) Zaghouan.

## Sample Availability

Samples are available on request to the corresponding author.
