# Supplementary material for: Screening for Astragalus hamosus Triterpenoid Saponins Using HPTLC Methods: Prior Identification of Azukisaponin Isomers
Source: Molecules. 2022 Aug 23;27(17):5376. doi: 10.3390/molecules27175376 (PMC9457977; doi:10.3390/molecules27175376)

Figure S1 : Four populations of *Astragalus hamosus* collected in Tunisia. Populations of (a) Bizerte, (b) Kairouan, (c) Siliana and (d) Zaghouan.

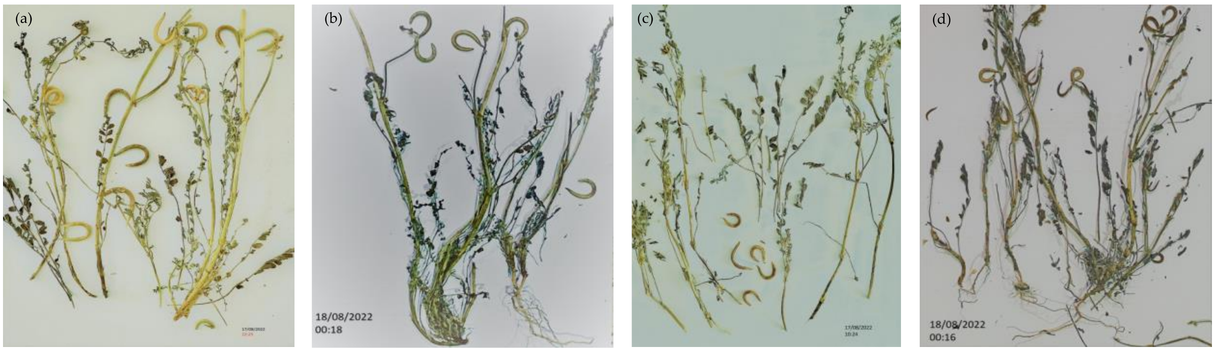

Supplement: Supplementary file 1 [file molecules-27-05376-s001.zip › molecules-1853835-supplementary.pdf]
